# Aversive Learning Deficits and Depressive-Like Behaviors Are Accompanied by an Increase in Oxidative Stress in a Rat Model of Fetal Alcohol Spectrum Disorders: The Protective Effect of Rapamycin

**DOI:** 10.3390/ijms22137083

**Published:** 2021-06-30

**Authors:** Malgorzata Lopatynska-Mazurek, Lukasz Komsta, Ewa Gibula-Tarlowska, Jolanta H. Kotlinska

**Affiliations:** 1 Department of Pharmacology and Pharmacodynamics, Medical University, 20-093 Lublin, Poland; gosia.lopatynska@gmail.com (M.L.-M.); ewa.gibula@umlub.pl (E.G.-T.); 2 Department of Medicinal Chemistry, Medical University, 20-090 Lublin, Poland; lukasz.komsta@umlub.pl

**Keywords:** Rapamycin, neonatal ethanol exposure, learning, depressive-like behavior, adult rats, oxidative stress

## Abstract

Fetal alcohol spectrum disorders (FASDs) are one of the most common consequences of ethanol exposure during pregnancy. In adulthood, these disorders can be manifested by learning and memory deficits and depressive-like behavior. Ethanol-induced oxidative stress may be one of the factors that induces FASD development. The mammalian target of the Rapamycin (mTOR) signaling pathway that acts via two distinct multiprotein complexes, mTORC1 and mTORC2, can affect oxidative stress. We investigated whether mTOR-dependent or mTOR-independent mechanisms are engaged in this phenomenon. Thus, Rapamycin—a selective inhibitor of mTORC1, Torin-2—a non-selective mTORC1/mTORC2 inhibitor, and FK-506—a drug that impacts oxidative stress in an mTOR-independent manner were used. Behavioral tests were performed in adult (PND60-65) rats using a passive avoidance (PA) task (aversive learning and memory) and forced swimming test (FST) (depressive-like behaviors). In addition, the biochemical parameters of oxidative stress, such as lipid peroxidation (LPO), as well as apurinic/apyrimidinic (AP)-sites were determined in the hippocampus and prefrontal cortex in adult (PND65) rats. The rat FASD model was induced by intragastric ethanol (5 g/kg/day) administration at postnatal day (PND)4–9 (an equivalent to the third trimester of human pregnancy). All substances (3 mg/kg) were given 30 min before ethanol. Our results show that neonatal ethanol exposure leads to deficits in context-dependent fear learning and depressive-like behavior in adult rats that were associated with increased oxidative stress parameters in the hippocampus and prefrontal cortex. Because these effects were completely reversed by Rapamycin, an mTORC1 inhibitor, this outcome suggests its usefulness as a preventive therapy in disorders connected with prenatal ethanol exposure.

## 1. Introduction

Fetal alcohol spectrum disorder (FASD) is the most severe consequence of prenatal alcohol exposure (PAE) during pregnancy. The most profound effects of PAE are neurobehavioral deficits, including physical and cognitive disabilities [1,2]. These effects are long lasting and have possible lifelong implications [3].

Such effects may be induced by the influence of ethanol on neurogenesis and on myelination of the central nervous system (CNS) during brain development [4]. A critical period of human pregnancy for brain development is the third trimester of human pregnancy (equivalent to the first 10 postnatal days (PND) in rodents) [5]. During this period, the prefrontal cortex and hippocampus, along with other brain regions, go through a transient period of mass neurodevelopment and rapid growth (growth spurt) [6,7,8]. Maternal alcohol consumption during this period leads, among other issues, to anatomical abnormalities, depressive-like behaviors, and learning and memory impairments in children [9]. 

The mechanisms responsible for FASD-induced neurodegeneration are multifaceted and complex. One such mechanism is oxidative stress [10,11]. The brain is particularly vulnerable to the production of reactive oxygen species (ROS), because 20% of total oxygen in the body is metabolized by the brain and it has a limited amount of antioxidant capacity [12]. Numerous studies also indicate that oxidative stress plays a major role in the etiology of depression, anxiety, and cognitive disorders [2].

The mTOR serine/threonine kinase is an enzyme that, among other effects, regulates cell growth and protein homeostasis via two distinct complexes, mTORC1 and mTORC2. As research suggests, chronic ethanol exposure activates mTOR and causes impairments of the autophagy [13,14]. This is a catabolic process that maintains homeostasis in cells and is activated by nutrient deprivation, metabolic stress, or exposure to toxic substances [15,16,17]. These factors lead to reactive oxygen species (ROS) stimulation and neuroapoptosis [18]. Selective inhibitors of mTORC1 and non-selective mTORC1/mTORC2 inhibitors could decrease ROS and may have a neuroprotective effect, especially for cognitive disorders. However, other compounds may have an impact on oxidative stress in an mTOR-independent manner [19].

The present study investigated the hypothesis that development of emotional learning deficits and depressive-like behaviors in adult rats exposed to ethanol during the neonatal period are a function of oxidative stress that, in turn, depends on the mTOR signaling pathway. To evaluate this hypothesis, we used Rapamycin—a selective mTORC1 inhibitor, Torin-2—a non-selective mTORC1/C2 inhibitor, and FK-506—an inhibitor of oxidative stress that acts independently to mTOR. To conduct such study, the male rat pups received these substances as pre-treatment before ethanol (5 g/kg/day, intragastrically) administration during PND4–9 so as to protect their brain cells against oxydative stress development. As adults (PND60), aversive learning and memory processes were tested by means of the inhibitory passive avoidance (PA) task [19,20,21,22], while depressive-like behavior was assessed using the forced swimming test (FST). Additionally, we evaluated the level of reactive oxygen species (ROS) by quantifying apurinic/apyrimidinic (AP)-sites and the production of LPO-malonodialdehyde (MDA) in the hippocampus and prefrontal cortex cells of the adult rats. It should be noted that AP sites are one of the major types of damage generated by ROS [23]. Similarly, the concentration of MDA in the tissues increases under the effect of increased ROS production [24]. ROS are generated during ethanol catabolism or from damaged mitochondria in response to ethanol exposure. At low levels, ROS are important regulators of cellular functions, but excessive ROS may cause oxidative stress, resulting in neurodegeneration characteristics for FASD [25]. Although most research into the issue of oxidative stress involves analysis in the first postnatal days [18,26,27,28], our work differs in that we investigated whether markers of oxidative stress are indentifiable in adult rats when behavioral deficits are present.

## 2. Results

### 2.1. Influence of Rapamycin, Torin-2, and FK-506 Pre-Treatment before Ethanol Administration during PND4–9 on Fear Learning and Memory as Measured in the PA Test in Adult (PND60/61) Male Rats

**Figure 1:**
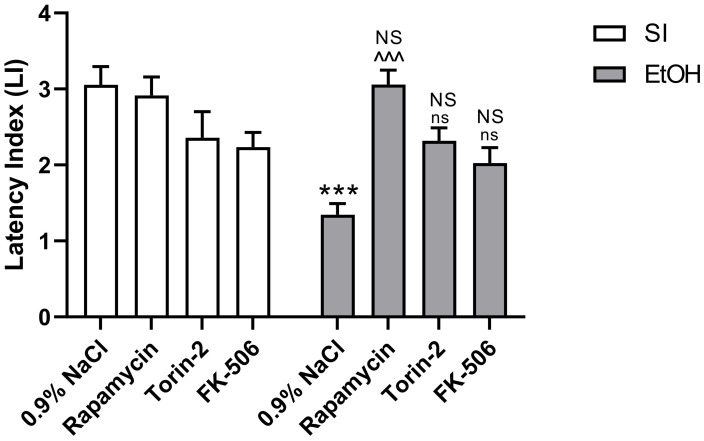
The influence of Rapamycin, Torin-2, and FK-506 pre-treatment (3 mg/kg, i.p.) during the neonatal period before ethanol administration (PND4–9) on fear learning and memory, tested 24 h after the training session, as measured by the PA task (latency index) in adult (PND61) male rats. Data are displayed as mean  ±  SEM of 8 subjects per group. *** *p* < 0.001 vs. SI; ^^^ *p* < 0.001, ns—nonsignificant vs. EtOH; NS—nonsignificant vs. the non-alcohol treated controls; SI—sham-intubation (i.g.); EtOH—ethanol intubation (i.g.).

In adult male rats, two-way ANOVA indicated a statistically significant effect of pre-treatment with the used substances (Rapamycin, Torin-2, and FK-506) (F(3,56) = 6.120; *p* < 0.01) and ethanol treatment (F(1,56) = 8.232; *p* < 0.01) on LI values of fear learning and memory and interaction between these factors (F(3,56) = 7192; *p* < 0.001). However, post-hoc analysis (Tukey’s test) revealed that only the ethanol-exposed group had significantly decreased LI values in rats in the PA task, compared to the control group (*p* < 0.001). This amnestic effect of ethanol was significantly reversed by Rapamycin pre-treatment at the dose of 3 mg/kg (*p* < 0.001), but not by Torin-2 and FK-506 pre-treatment at the dose of 3 mg/kg (*p* < 0.05) (Figure 1). Rapamycin, Torin-2, and FK-506 alone pre-treatment at the dose of 3 mg/kg also did not change the outcome of the undertaken experiments (*p* > 0.05).

### 2.2. Influence of Rapamycin, Torin-2, and FK-506 Pre-Treatment before Ethanol Administration during PND4–9 on Depressive-Like Behavior as Measured in the FST Test in Adult (PND65) Male Rats

**Figure 2:**
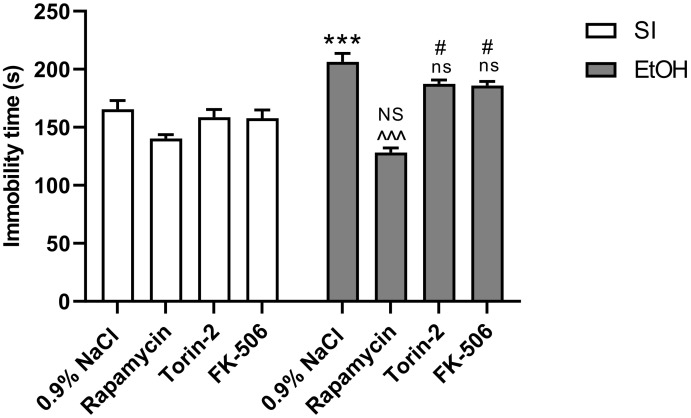
The influence of Rapamycin, Torin-2, and FK-506 pre-treatment (3 mg/kg, i.p.) during the neonatal period before ethanol administration (PND4–9) on the antidepressant-like effect in adult (PND65) male rats as measured by the forced swim test (FST). Data are displayed as mean ± SEM of 8 subjects per group; *** *p* < 0.001 vs. SI; ^^^ *p* < 0.001, ns—nonsignificant vs. EtOH; # *p* < 0.05, NS—nonsignificant vs. the non-alcohol treated controls; SI—sham-intubation (i.g.); EtOH—ethanol intubation (i.g.).

Two-way analysis of variance (ANOVA) indicated that there were statistically significant differences between pre-treated groups with the used substances (Rapamycin, Torin-2 and FK-506) (F(3,56) = 31.22; *p* < 0.001) and the ethanol treated groups (F(1,56) = 28.89; *p* < 0.001) on depressive-like behavior, as well as significant interactions between these two factors (F(3,56) = 8.352; *p* < 0.001). Post-hoc analysis (Tukey’s test) revealed that the ethanol-exposed group exerted a statistically significant depressive-like effect (*p* < 0.001). This was manifested in the reduction of total immobility time, in comparison to the control group. Pre-treatment with only Rapamycin had an anti-depressive effect *p* < 0.001). Torin-2 and FK-506 did not significantly decreased immobility time (*p* < 0.001). Rapamycin alone pre-treatment at the dose of 3 mg/kg did not change the outcome of the undertaken experiments (*p* > 0.05), but Torin-2 and FK-506 at the dose of 3 mg/kg did (*p* < 0.05) (Figure 2).

### 2.3. Influence of Rapamycin, Torin-2, and FK-506 Pre-Treatment before Ethanol Administration during PND4–9 on Locomotor Activity in Adult (PND59) Male Rats

Two-way analysis of variance (ANOVA) indicated that there were no statistically significant differences between pre-treated groups with Rapamycin, Torin-2, and FK-506 (F(3,56) = 0.6464; *p* > 0.05) at the dose of 3 mg/kg and ethanol-treated groups (F(1,56) = 3.921; *p* > 0.05) with regard to locomotor activity, and no significant interactions between these two factors (F(3,56) = 1.978; *p* > 0.05) (Table 1).

### 2.4. Influence of Rapamycin, Torin-2, and FK-506 Pre-Treatment before Ethanol Administration (PND4–9) on LPO Concentration in the Hippocampus and Prefrontal Cortex of Adult (PND65) Male Rats

Two-way analysis of variance (ANOVA) indicated the statistically significant effect of pre-treatment with Rapamycin, Torin-2, and FK-506 at the dose of 3 mg/kg on LPO concentrations in the hippocampus (F(3,56) = 3.880; *p* > 0.05) and prefrontal cortex (F(3,56) = 2.942; *p* > 0.05), and also the significant effect of ethanol on LPO concentrations in the hippocampus (F(1,56) = 95.08; *p* < 0.001) and prefrontal cortex (F(1,56) = 89.68; *p* < 0.001). Two-way analysis also indicated significant interactions between these two factors in the hippocampus (F(3,56) = 6.202; *p* < 0.01) and prefrontal cortex (F(3,56) = 6.926; *p* < 0.01). 

**Figure 3:**
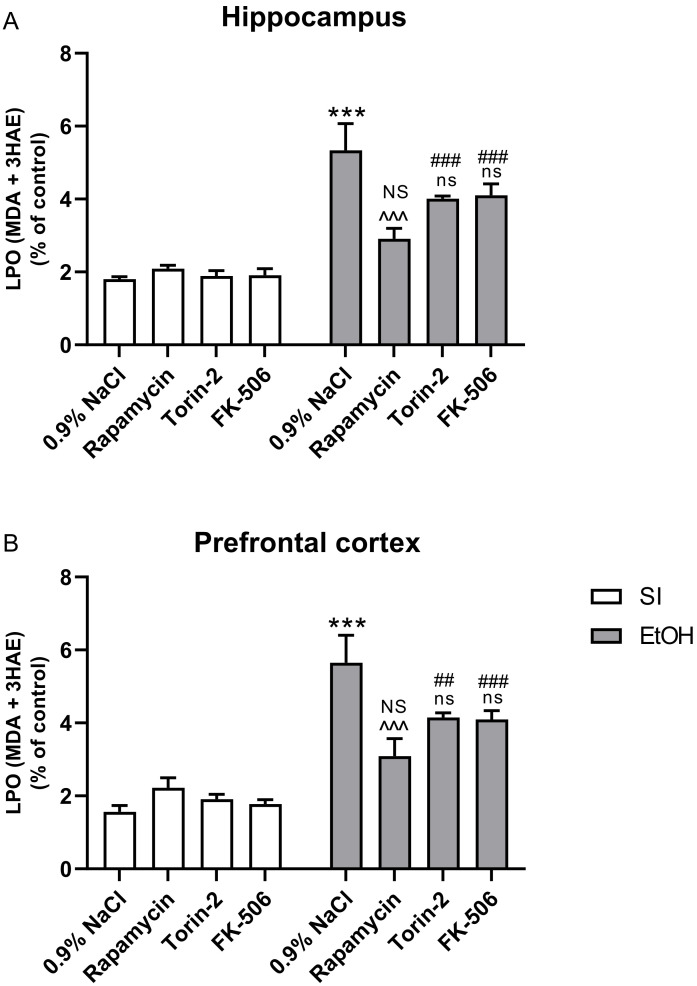
The influence of Rapamycin, Torin-2, and FK-506 pre-treatment (3 mg/kg, i.p.) during the neonatal period before ethanol administration (PND4–9) on LPO (MDA + 4 HAE) concentration in the hippocampus (**A**) and prefrontal cortex (**B**) of adult (PND61) male rats. The method is based on the measurement of the following products of lipid peroxidation: malondialdehyde (MDA) and 4-hydroxyalkenals (4HAE), which react with a chromogenic reagent N-methyl-2-phenylindole. Data are displayed as % of control (SI) mean ± SEM of 8 subjects per group, and are expressed as percentage of control group. *** *p* < 0.001 vs. SI; ^^^ *p* < 0.001, ns—nonsignificant vs. EtOH; ## *p* < 0.01, ### *p* < 0.001, NS—nonsignificant vs. the non-alcohol treated controls; SI—sham-intubation (i.g.); EtOH—ethanol intubation (i.g.).

Post-hoc analysis (Tukey’s test) in LPO concentrations in the hippocampus revealed that the ethanol-exposed group had increased ROS production (*p* < 0.001) compared to the control group. Pre-treatment with Rapamycin alone (*p* < 0.001) decreased this concentration, but pre-treatment with Torin-2 and FK-506 (*p* > 0.05) did not. The pre-treatment of Rapamycin alone at the dose 3 mg/kg also did not change the outcome of the undertaken experiments (*p* > 0.05), but pre-treatment at the dose 3 mg/kg with Torin-2 (*p* < 0.001) and FK-506 (*p* < 0.001) did (Figure 3A).

In LPO concentrations in the prefrontal cortex, post-hoc analysis (Tukey’s test) showed that the ethanol-exposed group had increased ROS production (*p* < 0.001) compared to the control group. Pre-treatment with Rapamycin alone (*p* < 0.001) decreased this concentration, but pre-treatment with Torin-2 and FK-506 (*p* > 0.05) did not. Rapamycin alone pre-treatment at the dose of 3 mg/kg did not change the outcome of the undertaken experiments (*p* > 0.05), but pre-treatment at the dose of 3 mg/kg with Torin-2 (*p* < 0.01) and FK-506 (*p* < 0.0001) did (Figure 3B).

### 2.5. Influence of Rapamycin, Torin-2, and FK-506 Pre-Treatment before Ethanol Administration (PND4–9) on AP Site Levels in the Hippocampus and Prefrontal Cortex of Adult (PND65) Male Rats

Two-way analysis of variance (ANOVA) indicated a statistically significant effect of pre-treatment with Rapamycin, Torin-2, and FK-506 on AP site levels in the hippocampus (F(3,56) = 9.930; *p* > 0.001) and prefrontal cortex (F(3,56) = 4.580; *p* > 0.01) and also the significant effect of ethanol treatment on AP site levels in the hippocampus (F(1,56) = 12.17; *p* < 0.001) and prefrontal cortex (F(1,56) = 8.353; *p* < 0.001). Two-way analysis also indicated significant interactions between these two factors in the hippocampus (F(3,56) = 68.55; *p* < 0.01) and prefrontal cortex (F(3,56) = 38.04; *p* < 0.01).

**Figure 4:**
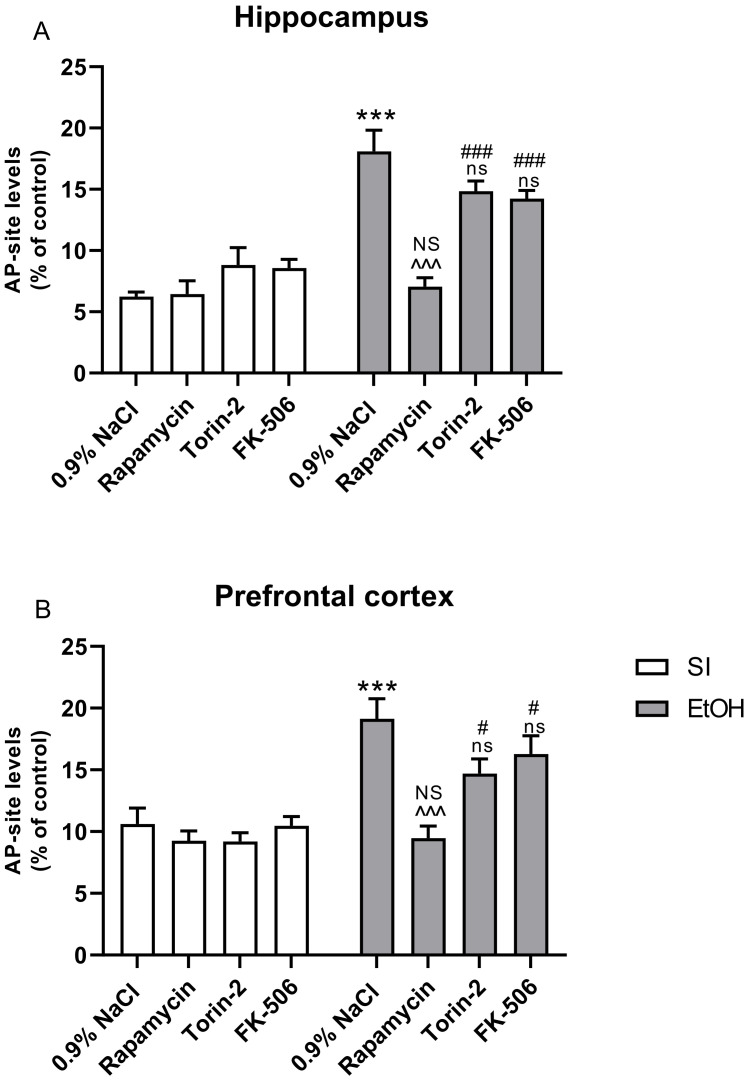
The influence of Rapamycin, Torin-2, and FK-506 pre-treatment (3 mg/kg, i.p.) during the neonatal period before ethanol administration (PND4–9) on oxidative damages of DNA in the hippocampus (**A**) and prefrontal cortex (**B**) of adult (PND61) male rats. The method is based on the measurement of simple basic sites (AP sites) in DNA. Data are displayed as **%** of control (SI) mean ± SEM of 8 subjects per group, and are expressed as percentage of control group. *** *p* < 0.001 vs. SI; ^^^ *p* < 0.001; ns—nonsignificant vs. EtOH; # *p* < 0.05, ### *p* < 0.001, NS—nonsignificant vs. the non-alcohol treated controls; SI—sham-intubation (i.g.); EtOH—ethanol intubation (i.g.).

Post-hoc analysis (Tukey’s test) in AP site levels in hippocampus showed that the ethanol exposed group had increased damage generated by ROS (*p* < 0.001), compared to the control group. Pre-treatment with Rapamycin (*p* < 0.001) decreased this AP site level and ROS generation, but pre-treatment with Torin-2 and FK-506 (*p* > 0.05) did not. Rapamycin alone pre-treatment at the dose of 3 mg/kg did not change the outcome of the undertaken experiments (*p* > 0.05), but Torin-2 and FK-506 pre-treatment at the dose of 3 mg/kg (*p* < 0.05) did (Figure 4A).

Post-hoc analysis (Tukey’s test) of AP site levels in the prefrontal cortex revealed that the ethanol-exposed group had increased damage generated by ROS (*p* < 0.001), compared to the control group. Pre-treatment with Rapamycin also decreased this AP site level and ROS generation (*p* < 0.001), but Torin-2 and FK-506 did not (*p* > 0.05). Moreover, Rapamycin alone pre-treatment at the dose 3 mg/kg did not change the outcome of the undertaken experiments (*p* > 0.05), but Torin-2 and FK-506 pre-treatment at the dose of 3 mg/kg (*p* < 0.05) did (Figure 4B).

## 3. Discussion

The current study demonstrated that the adult male rats exposed to ethanol during the neonatal period (PND4–9) showed behavioral deficits in fear learning and memory (according to the PA task results). Furthermore, our findings indicated that neonatal ethanol exposure induced depressive-like behavior in adult male rats according to the forced swimming test. Biochemical analysis also revealed an increase in LPO and AP sites in the prefrontal cortex and hippocampus of adult rats exposed to ethanol during the neonatal period. These data suggest that ethanol-induced cognition deficits and depressive-like behavior are related to increased oxidative stress in these brain structures during neonatal ethanol exposure. Pre-treatment with various mTOR-dependent or independent inhibitors of oxidative stress before ethanol administration during the neonatal period mitigated learning and memory impairment, prevented the depressive-like behavior, and attenuated the oxidative stress induced by ethanol. The most potent effects were observed after pre-treatment with Rapamycin, a selective mTORC1 inhibitor. Administration with this substance normalized these parameters to the values of the corresponding control groups and effectively prevented the harmful effects of ethanol on the developing brain.

### 3.1. Rapamycin Prevents Ethanol-Induced Fear Learning and Memory Impairments

Our study shows deficits in fear learning and memory in adult rats that received ethanol during the neonatal period. Thus, our results support the observations of other rat-based [29] and C57 mice-based studies, as well as studies of learning deficits in humans [30] prenatally exposed to alcohol. Generally, PA is a fear-motivated test classically used to assess long-term memory based on negative reinforcement in small laboratory animals (rat, mice). In our study, neonatal ethanol exposure significantly impaired all stages of contextual memory (acquisition, consolidation, and retrieval) in the PA task. Acquisition of this task requires the animals to learn to ignore their innate response to move to the dark chamber and instead to stay in the light side. Therefore, the task requires behavioral flexibility and inhibitory control (two behavioral features). However, we observed that ethanol-exposed animals required a significantly longer time to learn this task than control rats did. Published data show that emotionality (fear and/or anxiety) can [31] or cannot affect avoidance behavior [20]. However, our previous study indicated that neonatal ethanol-treated rats did not indicate anxiety-like behavior [32,33], therefore this factor could not have had influenced on the obtained results in the PA task.

Our findings indicate that Rapamycin, the mTORC1 selective inhibitor, given before ethanol administration during the neonatal period, reversed these ethanol effects. Furthermore, Torin-2—a non-selective mTORC1/C2 inhibitor—and FK-506—a substance that impacts oxidative stress in a mTOR-independent manner—did not prevent fear learning and memory impairments, although the results were partially lowered by these compounds. This means that Rapamycin pre-treated animals spent more time in the bright compartment and less entered the dark compartment where they had been previously shocked. Furthermore, our study confirms the participation of mTORC1 in this preventive mechanism and indicates that the non-selective mTORC1/C2 inhibitor and other substances without impact on mTOR pathway, do not show such behavior. Thus, our outcome suggests that only mTORC1 is engaged in FASD-induced learning and memory deficits. It should be noted that other factors such as ethanol-induced hyperlocomotion can affect the results in this task. However, increase in locomotor activity is restricted to adolescence because it was not observed in the adult (PND61) rats in our study (Table 1). This finding supports previous evidence [32,33,34] that hyperactivity is characteristic mostly for children with FASD [35] and becomes subtler when they become older.

### 3.2. Rapamycin Prevents Ethanol-Induced Depressive-Like Behavior

Various psychiatric illnesses, including depression, that are recognizable in young children and in adults [36,37] are a consequence of alcohol consumption during pregnancy and FASD development. Our study identified increased immobility in the forced swimming test, in adult rats exposed to ethanol during the neonatal period. The behavioral despair, which is assessed as immobility, is interpreted as a depressive-like behavior. These findings in neonatal ethanol-exposed rats confirm and extend those of others [38,39,40], who also reported that adult mice and rats that received ethanol during the neonatal period displayed increased immobility time in the forced swimming test. These observations are due to reduced locomotor activity in the ethanol-treated rats. However, our rats did not differ from non-alcohol control rats in their locomotor activity, further supporting the conclusion that behaviors in the forced swimming test represent depressive-like behaviors (Table 1). The reason for such discrepancies could be that our studies were conducted on male adult rats, but clinical depression is more frequently observed in female rats [39,41,42]. The choice of male rats in these experiments was based on our previous findings that showed no differences between the sexes in all the behavioral and biochemical studies in adult rats receiving ethanol during the neonatal period [32,33]. In the present study, Rapamycin, the selective mTORC1 inhibitor, given before ethanol administration during the neonatal period prevented these depressive-like behaviors in adulthood. Moreover, our study indicates that Torin-2, the non-selective mTORC1/C2 inhibitor, and FK-506, which impacts oxidative stress in an mTOR-independent manner, cannot prevent the depressive-like behaviors. Because Rapamycin is an mTORC1 inhibitor [43], our data emphasize the involvement of mTORC1 in depression in adults that is induced by neonatal alcohol exposure.

### 3.3. Rapamycin Prevents Ethanol-Induced LPO and Oxidative DNA Damage

Although Rapamycin possesses anti-inflammatory attributes, our study was focused on its anti-oxidant properties [44]. Thus, markers of oxidative stress were evaluated. Previous research has shown that neonatal ethanol exposure, which leads to FASD, is related to oxidative stress and increased ROS production [18]. The main source of ROS formation is the mitochondrial failure that is associated with neurodegeneration [45]. One of the most important consequences of ROS overproduction during FASD development is the modification of DNA that leads to cognitive and depressive-like disorders. For these reasons, the objective of our study was to evaluate oxidative DNA damage in the brain of rats submitted to neonatal ethanol exposure (PND4–9). Our study, conducted in adult rats, reveals that markers of oxidative stress induced by neonatal ethanol exposure are still present in rats of this age block. Thus, our results confirmed that of several other studies [46,47,48]. The current work reveals an increase in LPO in the hippocampus and prefrontal cortex of rats exposed to ethanol during the neonatal period, which indicates oxidative stress. This comes about because highly reactive oxygen metabolites act on the unsaturated fatty acids of phospholipid components of membranes to produce malondialdehyde, a LPO product. Reactivity of MDA and 4-HNE may cause damage to DNA [49]. In the brain, there is a significant amount of unsaturated fatty acids, which are susceptible to peroxidation. LPO decreases the life span of neurons, affects neurotransmitter release, and was reported as being a major contributor to the loss of cell function under oxidative stress conditions in depression and cognitive functions [50,51].

In ethanol-induced neurodegeneration affecting the brain, DNA damage induced by oxidative stress is also one of the major factors leading to neuronal dysfunction and cell death. Studies have shown that in brain structures such as the hippocampus and prefrontal cortex, a relationship exists between DNA damage and the presence of cortisol, especially during prolonged exposure to high levels of cortisol [52]. Oxidant-induced DNA damage may, therefore, be a useful biomarker for chronic oxidative stress determination [53]. Oxidative damage is also believed to contribute substantially to the decline in cellular functions that are associated with CNS diseases [54].

Previous studies have shown that oxidative DNA damage is linked to the onset of specific human diseases such as neuronal degeneration [53,54,55]. One of the major types of damage generated by ROS is at the AP site and results in increased levels of AP site markers within the LPO. This is the most common DNA damage that is the outcome of the loss of a DNA base [56]. Thus, our study revealed a statistically significant increase of AP site accumulation in the DNA and LPO isolated from the hippocampus and prefrontal cortex of rats that received ethanol during the neonatal period. Moreover, our results clearly show that pre-treatment with Rapamycin, a selective mTORC1 inhibitor, prevented the ROS accumulation in the hippocampus and prefrontal cortex, the brain regions related with FASD development. These results agree with previous findings showing that mTORC1 inhibitors alleviated ethanol-mediated ROS accumulation and attenuated ethanol-induced neuroapoptosis in cultured neurons and in the developing brain [18]. Our study also shows that Torin-2, the non-selective mTORC1/C2 inhibitor, and other substances such as FK-506 are without impact on the mTOR pathway and cannot prevent the ethanol-stimulated ROS accumulation.

### 3.4. Potential Mechanism

The exact mechanism of Rapamycin involvement in FASD development and potential protection still remains unknown. Oxidative stress, which is caused by expressive production of ROS, has been proposed as a one of the mechanisms for ethanol-induced neuronal damage and is associated with modulation of behavioral processes underlying the performance of cognition and emotions. To explore this hypothesis, we evaluated the effect of various modulators of mTOR so as to ascertain which pathway is involved in oxidative stress and inhibition, as well as which substance has potential therapeutic value. The present study confirms that FASD may be a result of oxidative stress. In our study, the level of LPO and AP site damage was increased in the hippocampus and prefrontal cortex, two brain regions involved in learning and memory and depression. Moreover, these data support the previous findings that prenatal ethanol exposure is associated with an increase in oxidative stress in the brain regions involved in neurodegeneration [57]. The effects we saw were ameliorated by Rapamycin but also by Torin-2 and FK-506. However, the results induced by Torin-2 (a non-selective mTORC1/C2 inhibitor) and FK-506 (which impacts oxidative stress in a mTOR-independent manner) administration did not have significant impact. Thus, we can suggest that neonatal ethanol exposure may induce deleterious effects in the mTORC1 signaling pathway and this leads to an increase in LPO and AP site production.

A recent study confirmed that oxidative stress in ethanol-treated rats brings about damage to mitochondria (essential in regulation of apoptosis), and results in the production of intracellular reactive oxygen [58]. Our data confirm the involvement of oxidative stress, but the limitation of the study is a lack of data on apoptosis and autophagy—which are also mTOR dependent. Other studies demonstrate that in experimental FASD models, cell loss is mediated by apoptosis, mitochondrial dysfunction, and impaired survival signaling through insulin and IGF-I receptor tyrosine kinases [59,60,61,62]. Yet another mechanism of FASD is the destruction of autophagy that offers protection against neurodegeneration. Rapamycin selectively activates autophagy (mitophagy) and eliminates the defective mitochondria. This could be the one of the mechanisms of Rapamycin that induces neuronal protection against ethanol-induced oxidative stress and neuronal death [63,64]. Such mechanism was also pointed out by other authors [18,25,65,66]. Moreover, previous work (including our own) indicates that the glutamatergic neurotransmission that takes part in brain development is affected by fetal/neonatal ethanol exposure and can be rescue by mTOR inhibitors [32,33].

Taken together, our findings suggest that Rapamycin, as a selective inhibitor of mTORC1, can act neuroprotectively in response to ethanol neurotoxicity, and that it alleviates ethanol-mediated oxidative stress and neuronal death. It is possible that the described changes are dependent on autophagy flux, hence, the exact mechanism of such interactions needs further evaluation. Overall, our work reveals that mTORC1 inhibitors play an important role in depressive-like behaviors and learning functions in adult rats that received ethanol during the neonatal period and may be useful as a preventive therapy in disorders connected with prenatal ethanol exposure.

## 4. Materials and Methods

### 4.1. Animals

Wistar rats (Medical University of Lublin, Lublin, Poland) were bred and housed in a vivarium under standard laboratory conditions: a light/dark cycle of 12 h/12 h, consistent temperature of 22 ± 1 °C, controlled humidity of 55 ± 10%, and free access to standard laboratory chow (Sniff Spezialdiäten GmbH, Soest, Germany) and water ad libitum. For breeding, one male and one female rat were housed together for one week. Beginning 3 weeks after mating, the females were checked twice a day for parturition. Following parturition, on PND3, male pups were assigned to double experimental groups (Experiment 1: behavioral paradigm, Experiment 2: biochemical analysis) 8 pups each and retaining equal numbers (see below). All rats (male and female) were housed with dams to PND21 then housed with 2–3 littermates through PND59. Our experiments used only male rats. This is because, according to prior research [32,67,68], male rats are more vulnerable to fetal/neonatal ethanbol exposure. All females were transferred to other studies. The rats were handled once a day for five days before the beginning of the behavioral experiments that were initiated at PND59. The biochemical experiments were conducted at PND65 after completing the behavioral part of study, in addition to any other studies involving the live animals. The experiments were conducted between 8:00 a.m. and 4:00 p.m. and carried out according to the National Institute of Health Guidelines for the Care and Use of Laboratory Animals, as well as the European Community Council Directive for Care and Use of Laboratory Animals (86/609/EEC) and were approved on 1 July 2019, by the Local Ethics Committee (54/2019).

### 4.2. Drugs and Neonatal Treatment

On PND3, male rat pups were paw-marked for identification and were assigned into 1 of 8 treatment groups that were used in the behavioral and biochemical experiments: 0.9%NaCl + sham intubated, Rapamycin + sham-intubated (3 mg/kg), Torin-2 (3 mg/kg) + sham-intubated, Fk-506 (3 mg/kg) + sham intubated, 0.9%NaCl + ethanol, Rapamycin (3 mg/kg) + ethanol, Torin-2 (3 mg/kg) + ethanol, or FK-506 (3 mg/kg) + ethanol (see Table 2). Induction of FAS was started on PND 4 and was carried out according to the method found in [32]. Ethanol (95% *w*/*v*, Polmos, Poznan, Poland) was given on PND4-9 as a “3rd trimester exposure” model. Pups received ethanol via intragastric intubation (i.g.) (5.0 g/kg/day; 22.66% *v*/*v*) delivered in a milk (Bebilon 1 Pronutra Plus) solution. This dose of ethanol produces significant neurotoxicity during the third trimester equivalent and may lead to neurobehavioral deficits [69]. The other substances, Rapamycin (Selleckchem, Munich, Germany), Torin-2 (Tocris Bioscience), and FK-506 (Tocris Bioscience), were dissolved in 0.9% NaCl and given intraperitoneally (i.p.), 1 h before ethanol intubation at the dose of 3 mg/kg. The Rapamycin dose was chosen based on a previous study by [70], the Torin-2 and FK-506 doses were chosen based on the approach used by [71]. Sham-intubated control rats received i.g. intubation without ethanol-milk or milk solution.

### 4.3. Experiments

#### 4.3.1. Passive Avoidance (PA)

The passive avoidance procedure produces similar results in both mice and rats [72]. The PA apparatus consisted of a two-compartment acrylic box with a lighted (8 W light) chamber (10 × 13 × 15 cm) and a darkened chamber (25 × 20 × 15 cm) equipped with an electric grid floor. The compartments were separated by guillotine door. Entrance of animals to the dark box was punished by an electric foot shock (0.15 mA for 2 s). 

The PA procedure was based on the association formed between an aversive stimulus (foot shock of 0.15 mA intensity and 2 s duration) and a specific environmental context (dark compartment). The experiment consisted of three steps: habituation (PND60), training (PND60), and retention testing (PND61). The procedure was as follows.

All rats were allowed to habituate in the experimental room for at least 30 min prior to the experiments. Next, in the training session, each animal was gently placed in the light compartment of the apparatus and allowed to freely explore. After 30 s, the guillotine door was opened, and the rat was allowed to enter the dark compartment. When the rat entered the dark chamber, the guillotine door was closed and the electric foot-shock duration was delivered immediately to the animal via the grid floor of the dark room by an insulated stimulator. Next, the animal was removed from the apparatus and placed in its home cage. The latency with which the animal crossed into the dark compartment was recorded (TL1). If the rat failed to enter the dark box within 300 s, it was placed into this dark box, the door was closed, and the electric foot-shock was delivered to the animal. In this case, TL1 value was recorded as 300 s. On PND61, 24 h later, the retention test trial was conducted with exactly the same conditions, but without electric shock. The latency with which the animal crossed into the dark compartment was recorded (TL2) and the time spent in the dark compartment was measured up to 300 s. If the animal did not enter the dark chamber within 300 s, the test was terminated and TL2 was recorded as 300 s.

To assess memory-related behaviors, the changes in the PA task were expressed as the difference between training and test latencies and were taken as the latency index (LI). LI was calculated for each animal as the ratio: LI = TL2-TL1/TL1, where: TL1—the time taken to enter the dark compartment during the training and TL2—the time taken to re-enter the dark compartment during the retention [73]. A high value of IL proves the positive influence of the tested substances on learning processes and memory, while low IL indicates disturbances in short-term memory processes and cognitive processes.

#### 4.3.2. Forced Swim Test (FST)

The experiment was carried out using the test proposed by [74] to examine the antidepressant effect of drugs. The main idea is based on the observation of an animal forced to swim in a situation without possibility of escape. After the initial period of vigorous movements, the animals give up further attempts (immobility time), which reflects a human sense of hopelessness. The longer the time of immobility, the greater the tendency of the animal to depressive behavior in comparison to the control group.

The FST procedure consisted of a habituation (PND64) and test phase (PND65). The procedure was as follows.

During the habituation phase, on the first day of the experiment, each rat was placed individually in a glass cylinder (10-liter capacity) filled with water (23–25 °C) for 15 min in order for it to adapt to the new conditions. After this time, the animals were returned to their home cages. On PND65, 24 h later, the test phase was conducted. The animals were placed individually in the water-filled cylinder again for 5 min and the total immobility time was measured by means of stopwatch. Immobility was assumed when the animal floated passively (in a semi-horizontal position), performing only enough movement necessary to keep its head above water (the natural behavior of animals is to try to swim and escape, alternating between being active and being motionless).

#### 4.3.3. Locomotor Activity (LA)

The locomotor activity of individual rats was recorded using a photocell apparatus (Porfex, Bialystok, Poland) and was undertaken to exclude the impact of locomotor on the PA and FST results. The apparatus was a square cage (60 × 60 cm^2^) made of transparent plexiglass and was designed for measuring locomotor activity (Porfex, Białystok, Poland). The cages were located in a sound-attenuated experimental room with constant lighting (40 W). The procedure was as follows, and was based on a method used in previous studies [32,33]:

The animals (PND59) were placed in the square Plexiglas cage. After 10 min of acclimatization, the total horizontal activity (distance traveled in meters) of individual animals was measured (15 min) by two rows of infrared light-sensitivity photocells placed 45 and 100 mm above the floor. To remove odor traces, the apparatus was thoroughly cleaned with water between each individual test run.

#### 4.3.4. Determination of Biochemical Parameters

On the last day of behavioral experiments (PND65), the animals were humanely killed and the prefrontal cortex and hippocampus were isolated from the rats’ brains. During the next step, tissue was homogenized from the frozen brain samples using extraction buffer. All biochemical measurements were conducted from these homogenates. The experimental procedures were performed according to the instructions supplied with each respective kit.

In this experiment, lipid hydroperoxide (LPO) testing was undertaken. LPO was based on malondialdehyde and 4-hydroxyalkenals concentration (MDA + 4HAE) (OxisResearch, Rockville, MD, USA). The principle underlying LPO assessment is based on the reaction of a chromogenic reagent R1 (N-methyl-2-phenylindole) with malondialdehyde (MDA) and 4-hydroxyalkenals (4HAE) at 45 °C. Two molecules of R1 react with one molecule of MDA or 4-hydroxyalkenals to form a chromophore with an absorbance maximum at 586 nm. Measuring the concentration of MDA in combination with 4-hydroxyalkenals in methane sulfonic acid was used as an indicator of LPO level.

#### 4.3.5. Determination of DNA Oxidative Damage

DNA was isolated with a Syngen DNA Mini Kit (Syngen, Wroclaw, Poland) according to the manufacturer’s protocol. The concentration and purity of the genomic DNA were measured using a NanoDrop MaestroNano Micro-Volume Spectrophotometer (Maestrogen Inc., Hsinchu City, Taiwan) and adjusted to 100 μg/mL in TE buffer. Oxidative DNA damage was evaluated by measuring the number of basic sites (the so-called AP) with a DNA Damage Quantification Kit (Dojindo, Kumamoto, Japan). Oxidative attacks by ROS on the deoxyribose moiety lead to the release of free bases from DNA, generating strand breaks with various sugar modifications and simple basic sites (AP sites). The aldehyde-reactive probe (ARP; N′-aminooxymethylcarbonylhydrazin-D-biotin) reacts specifically with an aldehyde group present on the open ring form of AP sites, making it possible to detect DNA modifications that result in the formation of an aldehyde group. Biotin-avidin-specific connection and horseradish peroxidase were used for colorimetric detection at 650 nm. AP sites were measured in the DNA isolated from the hippocampus and prefrontal cortex of the rats.

### 4.4. Statistical Analyses

The results were analyzed using the GraphPad Prism version 8.00 for Windows, GraphPad Software (San Diego, CA, USA). The statistical significance of drug effects and the behavioral and biochemical tests were analyzed by two-way analysis of variance (ANOVA) with repeated measures. This was followed Tukey’s post-hoc test. The results were presented as means ± standard errors of means (SEM) of values. A *p* value of less than 0.5 was considered statistically significant for all tests

## 5. Conclusions

Collectively, the present data demonstrate that the mTORC1 signaling pathway is responsible for the neurodegenerative effects of alcohol on brain development during the neonatal period. Moreover, these data reveal that such effects are associated with increased oxidative stress parameters in the hippocampus and prefrontal cortex. Our results also suggest that Rapamycin (a selective mTORC1 inhibitor), but not Torin-2 (a non-selective mTORC1/C2 inhibitor) or FK-506 (which has an impact on oxidative stress in a mTOR-independent manner), could have protective effects against the ethanol-induced LPO and AP site levels in the hippocampus and prefrontal cortex. Both of the latter are indicators of oxidative processes. However, a better understanding of the mTORC1 mechanism and oxidative stress are needed to produce effective therapy against neurotoxicity induced by neonatal ethanol exposure.

## Figures and Tables

**Table 1:** The influence of Rapamycin, Torin-2, and FK-506 pre-treatment (3 mg/kg, i.p.) during the neonatal period before ethanol administration (PND4–9) on locomotor activity in adult male rats (PND59). NS—nonsignificant vs. the non-ethanol treated controls.

Group	N	Distance Traveled (m) ± SEM
1. 0.9% NaCl + SI	8	52.87 ± 2.631 (NS)
2. Rapamycin + SI	8	48.39 ± 4.486 (NS)
3. Torin-2 + SI	8	50.89 ± 1.982 (NS)
4. FK-506 + SI	8	50.84 ± 2.036 (NS)
5. 0.9% NaCl + EtOH	8	50.15 ± 2.72 1(NS)
6. Rapamycin + EtOH	8	53.52 ± 0.650 (NS)
7. Torin-2 + EtOH	8	53.80 ± 0.927 (NS)
8. FK-506 + EtOH	8	55.93 ± 3.058 (NS)

NS—nonsignificant vs. the non-alcohol treated controls.

**Table 2:** Experimental groups. SI—shame intubation (i.g.); EtOH—ethanol intubation (i.g.).

Number of Groups	Intraperitoneally (i.p.) Injection	Intragastric (i.g.) Intubation	N
1.	0.9%NaCl	SI	8
2.	Rapamycin	SI	8
3.	Torin-2	SI	8
4.	FK-506	SI	8
5.	0.9%NaCl	EtOH	8
6.	Rapamycin	EtOH	8
7.	Torin-2	EtOH	8
8.	FK-506	EtOH	8

## Data Availability

The data presented in this study are available on request from the corresponding author.
